# Effectiveness of therapeutic massage for improving motor symptoms in Parkinson's disease: A systematic review and meta-analysis

**DOI:** 10.3389/fneur.2022.915232

**Published:** 2022-09-05

**Authors:** Zhiran Kang, Hua Xing, Qiang Lin, Fanchao Meng, Li Gong

**Affiliations:** ^1^Department of Tuina, Shanghai YueYang Hospital of Integrated Traditional Chinese and Western Medicine Affiliated to Shanghai University of Traditional Chinese Medicine, Shanghai, China; ^2^Department of Tuina, Shanghai General Hospital, Shanghai, China

**Keywords:** therapeutic massage, Traditional Chinese Medicine, manual therapy (MT), meta-analyse, Parkinson's disease

## Abstract

**Background:**

Parkinson's disease (PD) causes movement disorders [called motor symptoms (MS)], and motor dysfunction poses a great barrier to the quality of life. Although pharmacological therapy like levodopa can relieve the symptoms, it can also cause complications, such as psychosis, nausea, and dyskinesia. A therapy with more minor side effects is needed for PD. Therapeutic massages are the most commonly used forms of complementary and alternative medicine (CAM), but no systematic review and meta-analysis have focused on the efficacy of massage on PD.

**Objective:**

To evaluate the quality of evidence and efficacy of therapeutic massage for improving MS in PD.

**Methods:**

We independently searched four electronic databases, including Chinese National Knowledge Infrastructure (CNKI), MEDLINE/PubMed, Embase, and Cochrane Library, for randomized controlled trials (RCTs) about therapeutic massage and other available manual therapies improving MS in PD from January 1, 2012, to December 31, 2021 (recent 10 years). The main outcome measures were total effectiveness and the Unified Parkinson's Disease Rating Scale (UPDRS), including UPDRS total, II, and III. For the statistical analysis, the risk ratio, standard mean difference, and 95% confidence interval (CI) were used to calculate effect sizes between groups. To determine heterogeneity, statistical index *I*^2^ was used.

**Results:**

A total of 363 PD participants in seven RCTs and one randomized pilot-control study were included in this meta-analysis. The total effectiveness showed that therapeutic massage was more effective than the intervention of the control group for improving MS [ratio risk (RR): 1.33, 95% CI (1.14–1.55), *p* = 0.0002]. The UPDRS-III scores showed that massage improves motor function more than the control group [SMD = −0.46, 95% CI (−0.67, −0.24), *p* < 0.00001]. But we found that massage performed no better than the control group in improving daily life activities [SMD = −0.15, 95% CI (−0.40, 0.10), *p* = 0.23].

**Conclusion:**

Therapeutic massage was effective in improving MS in PD. It is suggested to be an appropriate form of CAM in treating PD.

**Systematic Review Registration:**

https://www.crd.york.ac.uk/PROSPERO/display_record.php?RecordID=323182, identifier: CRD42022323182.

## Introduction

Parkinson's disease (PD) is a neurodegenerative disorder that progressively damages multiple organ systems in the human body. In Europe, the annual incidence of this disease ranges from 13.5 to 18.8 per 100,000. Therefore, the common opinion is that the prevalence of PD ranges from 1 to 2 per 10,000 (1, 2). Besides its high prevalence, PD is more likely to happen among the elderly, especially those aged above 60 years (3). Dysfunction of the neuromuscular system is one of the system damages caused by PD. The main pathological mechanism of the dysfunction is that dopamine in the striatum decreases because of degeneration of the dopaminergic neurons of the substantia nigra pars compacta (4). The declined function of neurons and muscles leads to motor symptoms (MS), mainly including tremors, bradykinesia, stiffness, and postural instability (5). These symptoms greatly impact patients' quality of life (QoL) (6).

Nowadays, the mainstream therapy for PD is drugs. As first-line treatments for MS in PD, monoamine oxidase B inhibitors, levodopa, dopamine agonists, and amantadine have definite effectiveness (7). However, patients still cannot maintain complete autonomy with pharmacological treatment only (8). Furthermore, adverse events are common problems brought by medications, such as dyskinesia (a series of motor complications) by levodopa treatment, somnolence by dopamine agonists, psychosis by anticholinergic drugs, and so on (9). Rehabilitative therapy is considered a good approach to help patients' QoL, yet it is still limited (10). In this context, a therapy with fewer side effects is needed to complement the treatment of MS in PD.

Therapeutic massage is a popular form of complementary and alternative medicine (CAM) worldwide (11, 12). Thai massage, Japanese massage, Traditional Chinese Tuina, and some other manual therapies have clinical records demonstrating their ability to improve motor function in PD patients. A study showed that the Anma massage (a Japanese massage) increased the motor ability of upper and lower limbs by improving gait function and movement range of shoulder joints (13–15). Also, upper limb strength was improved by a Thai massage (16). An osteopathic manipulation can also ameliorate sensory function and falls prevention function (17). Some studies about Chinese Tuina manipulations showed that they could improve movement ability, as demonstrated by reduced scores on the Unified Parkinson's Disease Rating Scales (UPDRS), especially UPDRS-III scores (18–22). Although a literature review reported that five massage techniques could enhance the treatment of both motor and non-MS, there is still a lack of systematic and quantitative review or meta-analysis to summarize the effective forms of manual therapy and analyze their definite efficacy for MS. Also, potential mechanisms of massage therapy have not yet been discussed (23). This study aimed to evaluate the quality of evidence and efficacy of therapeutic massage for improving MS in PD.

## Methods

### Search strategy

This systematic review is registered with the PROSPERO database (CRD42022323182). This systematic review and meta-analysis followed the preferred reporting items for systematic reviews and meta-analyses (PRISMA) statement principles, using the population, intervention, control, and outcomes (PICO) model. Two reviewers (ZRK and HX) independently searched the following electronic databases (one Chinese database and three databases in English): Chinese National Knowledge Infrastructure (CNKI), MEDLINE/PubMed, Embase, and Cochrane Library. Searching terms for all the databases are displayed in Supplementary Tables 1–4. Then they checked titles and abstracts independently to filter studies satisfying the retrieval strategies from January 2012 to December 2021 without language limitations. Other authors selected all relevant articles and reached a consensus by discussion.

### Study eligibility

Selected studies fulfilled the following inclusion criteria: (1) randomized control trials (RCTs) or randomized pilot studies, (2) certainly diagnosed PD participants with MS, (3) the experimental group including the intervention of manipulation therapy alone, (4) the control group that received basic clinical care, rehabilitative therapy, or basic drug therapy while excluding any manual therapy, and (5) assessment mainly including UPDRS-III, then UPDRS total and II.

### Data extraction and quality assessment

Based on our design, the characteristics and data of our study were assessed and extracted. Two authors (ZRK and QL) independently extracted the following data from selected studies: first author and year, country, study type, patients' characteristics (age, gender, amount, H&Y stage, and disease duration), the protocol of experimental and control groups, and outcome measures.

Two authors (ZRK and HX) independently assessed the quality of each selected study using version 2 of the Cochrane risk-of-bias tool (ROB-2) outlined in Chapter 8 of the *Cochrane Handbook for Systematic Reviews of Interventions (V6.3)* for risk of bias (24). The bias assessment was shown in five domains: randomization process, deviations from the intended interventions, missing outcome data, measurement of the outcome, and selection of the reported result. The overall risk-of-bias judgment is divided in three levels: low risk of bias, some concerns, and high risk of bias.

### Data synthesis and analysis

The analysis was run on the Cochrane Review Manager software v.5.4 (the latest version). UPDRS scores (UPDRS total, II, and III) were considered continuous data. We analyzed continuous data based on the standard mean difference (SMD). The total effectiveness was dichotomous data. For evaluation of dichotomous data, we used risk ratio (RR) and 95% confidence interval (CI). *p*-value < 0.05 represented statistical significance.

For the test of heterogeneity, *p* < 0.1 represented heterogeneity between studies that had statistically significant differences. *I*^2^ tests were assessed for all outcomes in our study. We regarded *I*^2^ ≥ 75 as significant heterogeneity, *I*^2^ ≤ 50 as low heterogeneity, and *I*^2^ < 75 but >50 as moderate heterogeneity. When the level of heterogeneity was moderate or low, we used a fixed-effects model. Otherwise, we used a random-effects model. If the heterogeneity was high, we prepared to run sensitivity analysis and explained the potential reasons for heterogeneity. A funnel plot was established to explore possible publication biases if more than ten studies were in the analysis. Egger's test was used to test the asymmetry of the funnel plot (25). We performed sensitivity analysis by putting aside one study at a time to ensure the robustness of the results.

## Results

### Study selection

**Figure 1:**
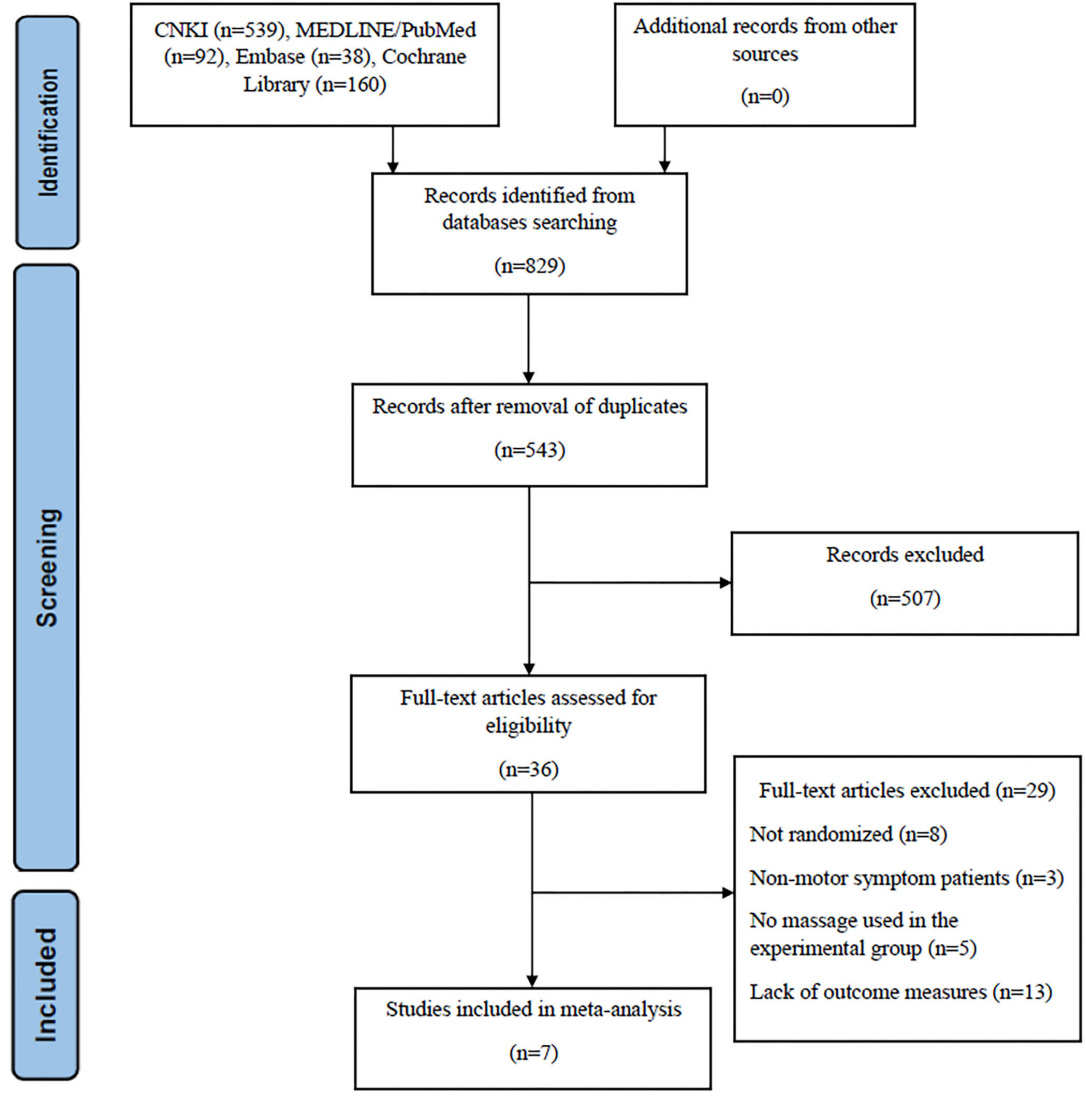
The 2020 version of the PRISMA flowchart showing the selection of included studies.

In the initial database search, we detected 829 studies. After removing duplicate studies and any irrelevant articles by screening titles and abstracts, 36 studies were left for full-text checking. When reading the full text of these articles, 29 were excluded. Of them, 8 were not randomized trials or even simply case reports; three of them focused on non-motor symptom patients, which did not match our study interest. A total of 5 studies did not use massage alone, and 13 studies lacked the outcome measure, UPDRS score. At the end of the selection process, 7 studies met the inclusion criteria (16, 18, 19, 21, 22, 26, 27) (Figure 1).

**Figure 2:**
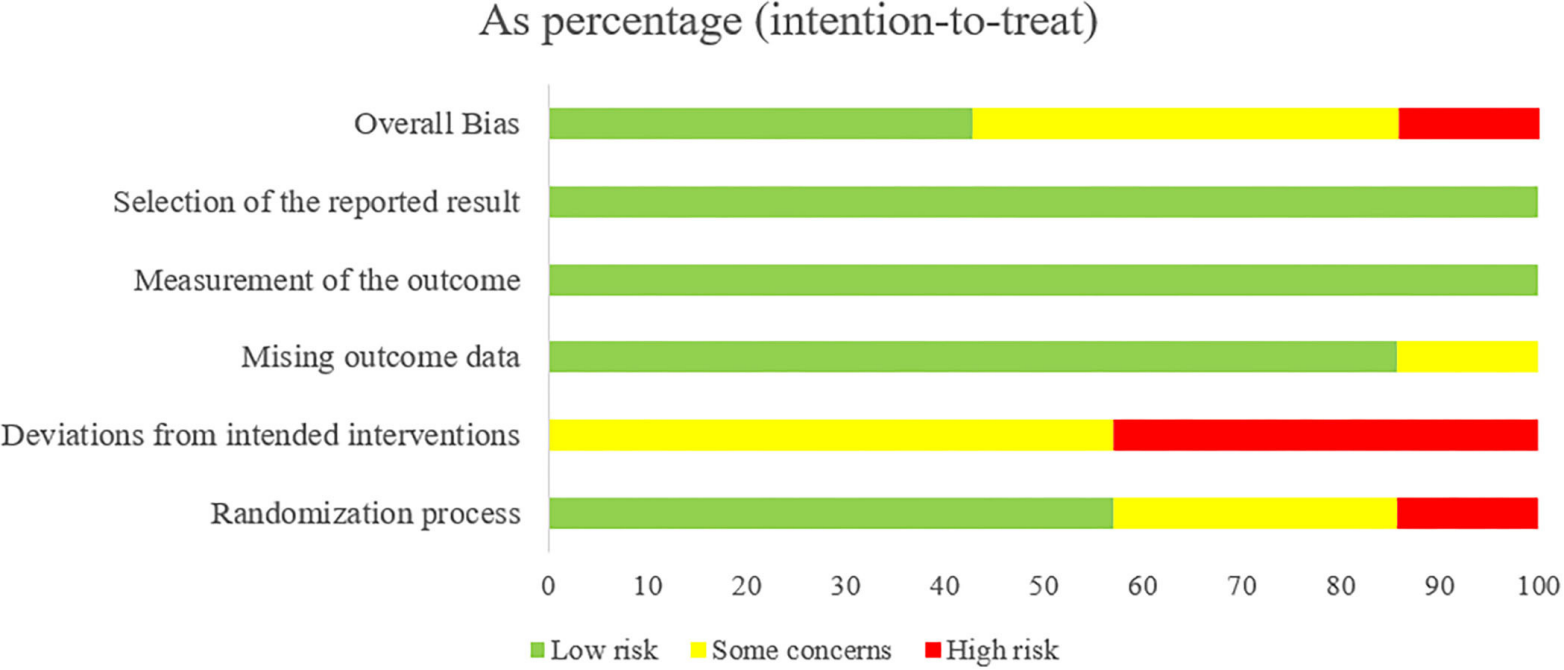
Risk of bias summary: review authors' judgments of each risk of bias item presented as percentages across all included studies.

**Figure 3:**
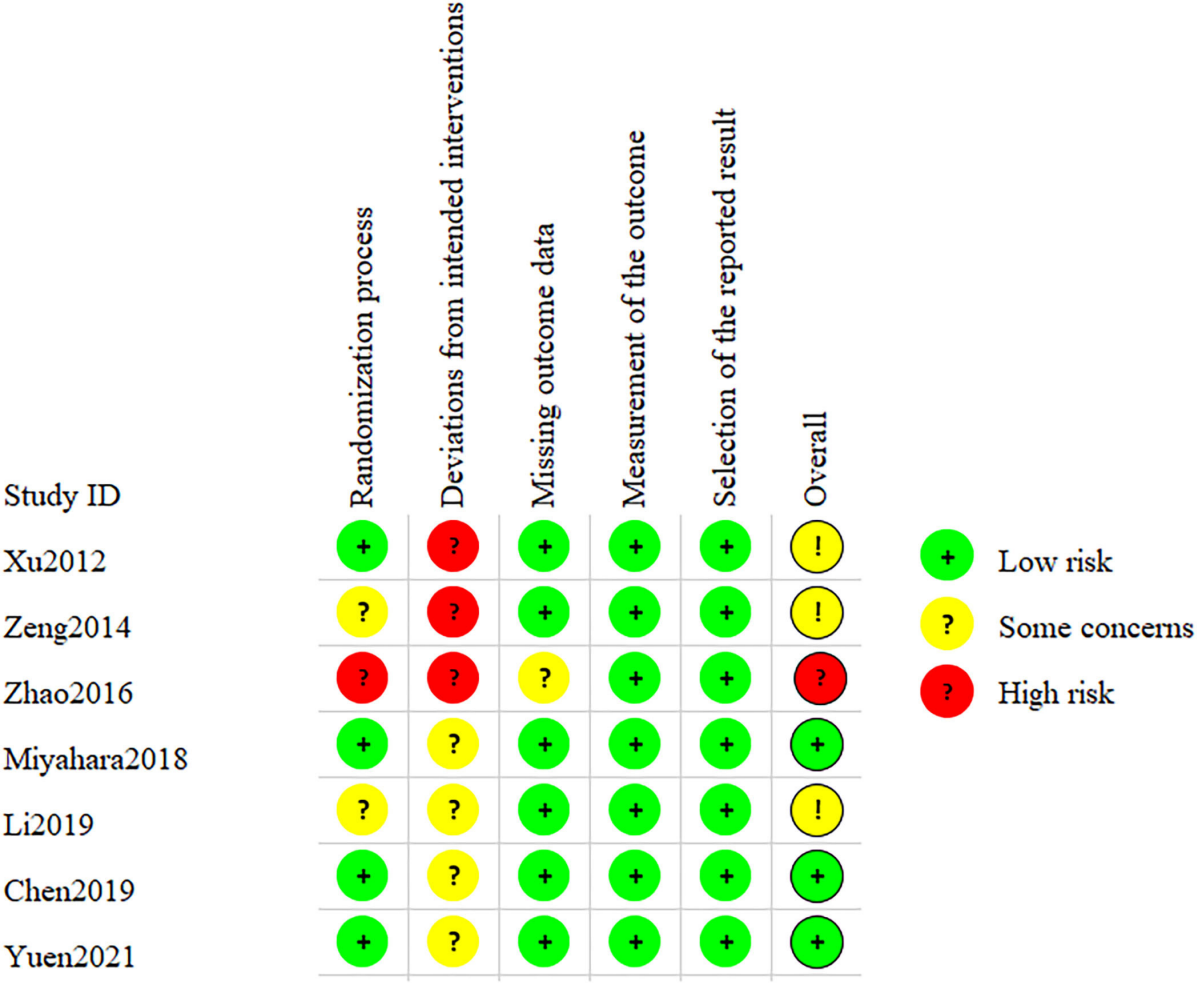
Risk of bias of studies: review authors' judgments of each risk of bias item for all included studies.

After assessing the Cochrane tool ROB-2, the condition of studies' quality came out. Figure 2 displays the overall quality of studies, which showed approximately 42.9% low risk, 42.9% some concerns, and 14.3% high risk of overall bias. Regarding bias of deviations from intended interventions, we had to consider that therapeutic massage is a technique requiring therapists to contact patients' bodies with their hands; complete blinding of participants and personnel was impossibly done. All included studies perform well on the bias of missing outcome data and measurement of the outcome. As shown in Figure 3, we saw that some studies had several items of high risk. Comparatively, Chen et al. and Yuen et al. (22, 27) got a good performance on risk of bias, which had five items of low risk, while Xu et al. and Zeng et al. (18, 19) had one high-risk item, and Zhao et al. had two high-risk items each (26).

### Patient characteristics

There were 363 PD participants with MS in 6 RCTs and 1 randomized pilot trial (Supplementary Table 5). In total, 182 people were from the experimental group, who received therapeutic massage, and the remaining 181 were from the control group, accepting basic clinical care, drug use, and health education. Also, 207 participants were male, while 156 participants were female. In terms of age, Chen et al.'s study had the oldest participants, in which the experimental group age was 82.18 ± 5.70, and the control group age was 83.63 ± 5.27 (22). Xu et al.'s study had the youngest participants; the experimental group age was 55.70 ± 7.08, and the control group age was 56.55 ± 6.99 (18). The disease duration in all included studies was not more than 10 years, but Zhao et al. did not display patients' duration of disease (26). As for the severity of PD, 5 studies chose a range from H&Y I–III. Li et al. did not mention severity (21), and Zhao et al.'s study included H&Y IV patients (26).

### Intervention characteristics

All included studies used therapeutic massage in the experimental groups (Supplementary Table 6). Three performed Traditional Chinese Tuina (18, 19, 21), a massage technique guided by ancient Traditional Chinese Medicine (TCM); two studies used common massage, targeting on limbs of patients (22, 26). Yuen et al. used acupressure, a technique in which the therapist performed pressing manipulation on specific acupoints. Miyahara et al. used Thai massage (16). As for the control group, two studies applied health education (18, 27), another two used clinical care (16, 22), and three studies took first-line pharmacological therapy of PD (19, 21, 26). However, one of them did not give details of the drug (26).

The duration of a single treatment session was <60 min but more than 20 min. Particularly, one study did not describe the duration of a single treatment session and the frequency (18). Three studies performed a massage every day of the week (19, 21, 27), while the lowest frequency was twice a week (16). The total therapy duration of most studies ranged between 1 and 2 months, but one study applied a long-term treatment (1 year) (26). Most studies only made an instant observation after treatment rather than at follow-up, except for one study that completed an 8-week follow-up (22).

### Efficacy of therapeutic massage: Total effectiveness

**Figure 4:**
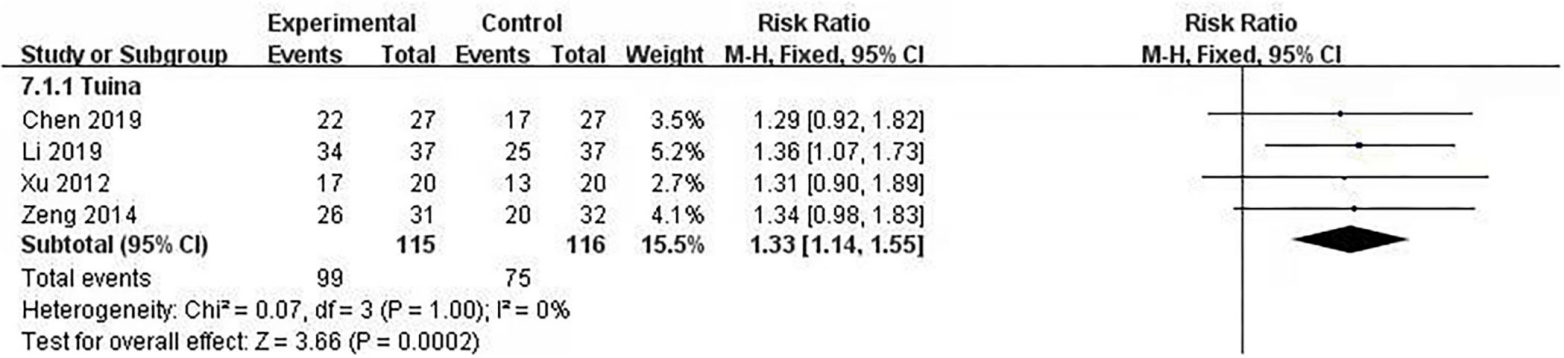
Comparison 1: therapeutic massage vs. control. Outcome 1: Total Effectiveness.

Total effectiveness is a way for clinical study to judge the effect of the Traditional Chinese Medicine intervention. It is divided efficacy into four levels: cured, highly effective, moderately effective, and poorly effective. Total effectiveness included the first three levels, and the calculation formula was $\left(\frac{\text{cured}+\text{highly effective}+\text{moderately effective patients}}{\text{total patients}\times 100\%}\right)$, which was based on the *Criteria of Diagnosis and Therapeutic Effect of Diseases and Syndromes in Traditional Chinese Medicine* (28). Four studies (231 patients included) used total effectiveness (18, 19, 21, 22) to explore the efficacy of interventions, and we put the post-intervention data into a meta-analysis. The meta-analysis showed no significant heterogeneity (*I*^2^ = 0%, *p* = 1.00). The fixed model was used. The results showed that the differences between therapeutic massage groups and control groups were statistically significant [RR = 1.33, 95% CI (1.14, 1.55), *p* < 0.05] (Figure 4). Sensitivity analyses implied no significant change when any study was removed.

### Motor function: UPDRS-III score

**Figure 5:**
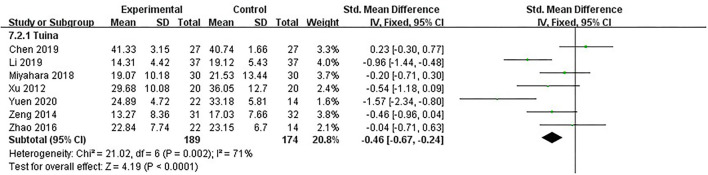
Comparison 2: therapeutic massage vs. control. Outcome 2: UPDRS-III.

UPDRS-III was the primary measure of our study. It is the third part of UPDRS, made up of 14 items, each scored “0–4,” for evaluating motor function. The evaluation covered speech function, MS of head and face, upper and lower limb function, tremor condition, gait, and instability of body control. All seven studies, including 363 patients, used UPDRS-III to assess motor function of PD patients (16, 18, 19, 21, 22, 26, 27). The heterogeneity between studies was moderate (*I*^2^ = 71%, *p* = 0.002). We used the fixed model. The results showed that after analyzing post-intervention data, therapeutic massage was significantly more effective than the control in improving MS [SMD = −0.46, 95% CI (−0.67, −0.24), *p* < 0.00001] (Figure 5). Sensitivity analyses implied no significant change when any study was removed.

### Overall condition: UPDRS-total score

**Figure 6:**
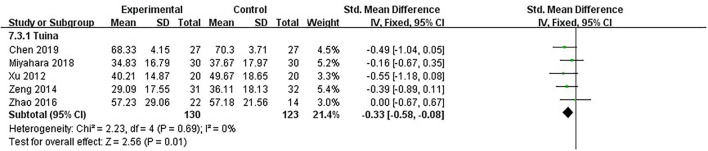
Comparison 3: therapeutic massage vs. control. Outcome 3: UPDRS-total.

The UPDRS-total score is the sum of UPDRS parts I, II, III, and IV scores, reflecting the overall condition of PD patients after treatment. The lower the score was, the better the efficacy was. Five studies, including 253 patients, used UPDRS-total as the assessment (16, 18, 19, 22, 26). Meta-analysis showed no significant heterogeneity in the included studies (*I*^2^ = 0%, *p* = 0.69); we used the fixed model. The differences were statistically significant between the therapeutic massage and control groups, implying that therapeutic massage was more effective in improving the overall condition of PD patients [SMD = −0.33, 95% CI (−0.58, −0.08), *p* < 0.05] (Figure 6). Sensitivity analyses implied no significant change when any study was removed.

### The function of daily living: UPDRS-II score

**Figure 7:**
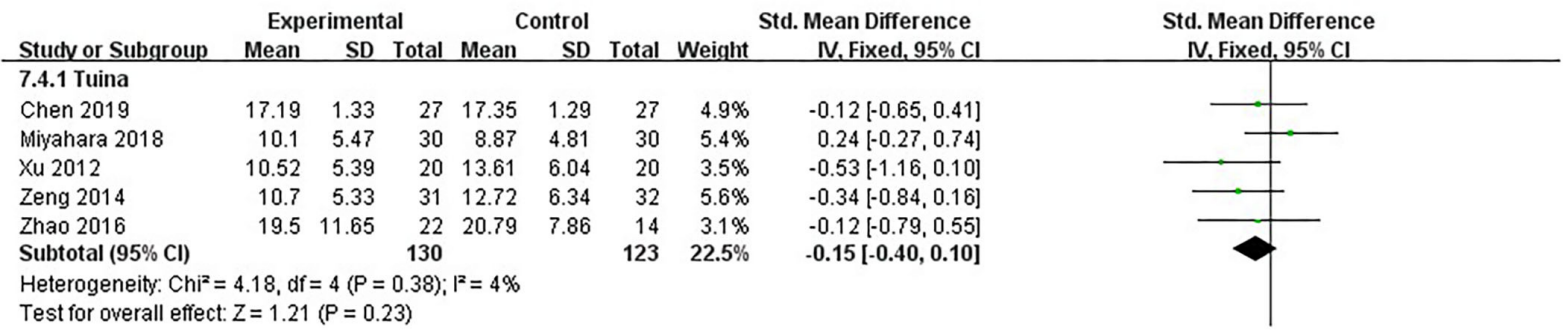
Comparison 4: therapeutic massage vs. control. Outcome 4: UPDRS-II.

UPDRS-II was the secondary measure of our study. It comprises 13 items, and although it mainly reflects daily life behavior, most of these behaviors were closely related to motor function. For example, the item “chewing and swallowing” needed the complex coordination of facial and pharyngeal muscles. The item “handwriting” was related to the motor function of the upper limbs and eyes. Therefore, UPDRS-II could be indirect evidence for assessing motor function. Five studies, including 253 patients, used UPDRS-II as the assessment (16, 18, 19, 22, 26). The heterogeneity between studies was little (*I*^2^ = 4%, *p* = 0.38); we used the fixed model. After analyzing post-intervention data, there were no significant differences in daily living improvement between the therapeutic massage and control groups [SMD = −0.15, 95% CI (−0.40, 0.10), *p* > 0.05] (Figure 7). Sensitivity analyses implied no significant change when any study was removed.

## Discussion

The systematic review and meta-analysis evaluated the efficacy of therapeutic massage for improving MS in PD by total effectiveness and UPDRS series score. After the screening and selecting process on four major literature databases, seven studies met the inclusion criteria, and we included them in the meta-analysis. The publication year of all studies was from January 2012 to December 2021 (recent 10 years). There were six RCTs and one randomized pilot study. The intervention of the experimental group was therapeutic massage, while that of the control group was basic drug therapy, clinical care, and health education. The test of homogeneity and several statistical tests were run in the meta-analysis. In total, 363 people with PD were included.

To evaluate the motor function and symptoms in included PD patients, UPDRS-III became the primary outcome measure (POM); we regarded total effectiveness, UPDRS-total, and UPDRS-II as the secondary outcome measures for evaluating the efficacy and other aspects of the effect of therapeutic massage. All seven included studies used the UPDRS-III score to assess motor function (16, 18, 19, 21, 22, 26, 27). Four studies used total effectiveness to assess the efficacy of interventions (18, 19, 21, 22); five used UPDRS total and UPDRS-II to assess the overall condition and daily living function of PD patients (16, 18, 19, 22, 26). Most of the studies did not have a follow-up except for one study, which had an 8-week follow-up (22).

The meta-analysis results showed that therapeutic massage had significantly better efficacy than the control group (*p* < 0.05). It is important to mention that the standard of levels division of total effectiveness was based on the POM that the study chose and the calculation between baseline and post-intervention scores. The calculation formula of the standard was $\left(\frac{POM_{baseline}-POM_{post}}{POM_{baseline}}\right)\times 100\%$. A percentage ≥85% was defined as clinically cured, a percentage ≥50% but <85% was defined as highly effective, a percentage ≥20% but <50% was defined as moderately effective, and a percentage <20% was regarded as poorly effective (28). Then, we could calculate total effectiveness based on the number of patients at each level. One of the four included studies used Webster Scale as the primary outcome (19) for calculating total effectiveness, while the other three used UPDRS (18, 21, 22). These four studies were all conducted in China, and most of them performed Traditional Chinese Tuina. This method of evaluating the efficacy of TCM therapy is popular in Chinese clinical trials rather than in other areas worldwide. However, its accuracy and comparability would be influenced by the choice of POM. Fortunately, the Webster Scale, which the study in our study used, was a 10-item scale and the content of all the items was related to motor function, which was similar to UPDRS-III. To sum up, researchers could not guarantee the consistency of the POMs used in their total effectiveness. We, therefore, do not recommend that researchers use total effectiveness in their clinical trials. If necessary and unavoidable, a unified standard measure was used to calculate total effectiveness as much as possible.

Results of the meta-analysis showed that therapeutic massage was more effective in improving the UPDRS-III score than the control intervention (*p* < 0.05), indicating that therapeutic massage could alleviate MS and improve motor function. The curative mechanism of massage was broadly discussed. During manipulation techniques performed on muscles of different body parts, it was proved that massage had a positive effect on muscle characteristics, including strength, tension, and extensibility, in several massage clinical trials on other diseases (29–32). Gait improvement was also commonly reported in massage intervention studies. It was proved when stimulating neck muscles; the velocity and direction of gait improved in patients with gait instability (33, 34), which is likely related to the activation of lower limbs muscles and the enhancement of proprioception (35). Refocusing on the performing details of massage in our included studies, we found that manipulating the limbs and trunk was a key process. Zhao et al. performed massage on the paraspinal muscle by thumb and thenar eminence of the hand, which created the effect of muscle relaxation and anti-rigidity (26). Miyahara et al. performed a very meticulous process of Thai massage (16). The therapist manipulated the fingers, palms, wrist, and inner and outer arms through the distal limbs to the proximal limbs. Acupoints on the arms were also pressed during the process, and finally, they applied grasping manipulation to the patients' shoulders and interscapular area. Besides limbs, Yuen et al. and Chen performed massage on the head and face of patients, especially on the muscle responsible for masticating, expressing, and swallowing, which helped improve the function of swallowing and speech (22, 27). The three included studies that used Traditional Chinese Tuina valued the application of TCM theory through the Tuina performed (18, 19, 21). TCM theory guiding Tuina therapy was mainly Meridian, Channel, and Acupoint theory. We TCM thought an essential substance called “Qi” flowed and moved in meridians and channels. The flow of “Qi” made up essential functions of the human body, such as motor function. Many functions are affected when the flow of “Qi” is blocked or deficient. Acupoints, which were gates regulating flow of “Qi” running from inside to outside and were also reflection spots of diseases or trigger points of Tuina therapy, were on the route of meridians and channels. Thus, we could use massage to contact meridians and acupoints to regulate the flow of “Qi”; then, the dysfunctional situation would be improved. For example, Li et al. used finger-pushing manipulation on EX-HN 3 and DU 24 of the frontal head, SP 10 and ST 36 of the lower limbs, LI 11 and SJ 5 of the forearms, and BL 18 and BL 23 of the lower back, which significantly improved motor scores of UPDRS after treating and improved more than the control group's score. That was why manipulating specific body parts could recover corresponding motor functions from the point of TCM view.

After the UPDRS-II score was meta-analyzed, results showed no significant difference between therapeutic massage and intervention of the control group for improving the daily living of PD patients (*p* > 0.05). The activities of daily life need the support of the motor function. The complex movement of the body makes up our life behavior. Interestingly, in comparison with the control group, therapeutic massage improved motor function well while hardly improving the quality of daily living. This phenomenon implied that improving motor function by therapeutic massage could not help PD patients perform their daily tasks well, making us view it rationally. After all, massage was a complementary therapy for diseases, and from this aspect, we thought it could not be the sole main therapy for PD. The direction of clinical trials may explore how to apply massage assisting first-line or regular therapy in enhancing motor function and daily life or letting massage mediate the reduction of adverse events of PD drugs.

In summary, therapeutic massage had relatively good efficacy for PD and for improving MS in PD to some extent. However, therapeutic massage did not have a satisfactory effect on the quality of daily life compared with the control group. Additionally, the heterogeneity of UPDRS-III between the two groups was comparatively high (*I*^2^ = 71%). We conducted a sensitivity analysis by removing one study at a time, but the level of heterogeneity did not change a lot. Restricted to the amount of data, we did not perform a subgroup analysis, but we inferred that the age of patients and severity could be the source of heterogeneity. Chen et al. enrolled patients in the community whose ages were beyond 80 years, while the other studies enrolled PD patients with an average age of 65 years. Older aging patients probably performed worse than younger patients (36, 37). Another possible source of heterogeneity was the severity of PD. Zhao et al. reported patients with H&Y stage IV, in which description was “the need for an assistive device or a person to help walk.” If H&Y stages are too high, there may be difficulty in treatment. Furthermore, more follow-up is needed in future studies. We should not only focus on the instant effect of massage but also care about its long-term efficacy.

## Conclusion

Therapeutic massage improved the overall condition and motor function better than the control intervention in terms of the total effectiveness, UPDRS-III, and UPDRS-total, but not UPDRS-II. More standardized and normalized RCTs are needed to make the meta-analysis more accurate and valuable.

## Data availability statement

The original contributions presented in the study are included in the article/Supplementary material, further inquiries can be directed to the corresponding author/s.

## Author contributions

ZK made substantial contributions to the conception or design and data acquisition of the study. HX made a contribution to data analysis and interpretation. ZK and LG drafted the manuscript or revised it critically for important intellectual content. HX, QL, and FM agreed to be accountable for all aspects of the study in ensuring that questions related to the accuracy or integrity of any part of the work are appropriately investigated and resolved. LG approved the final version to be published. All authors contributed to the article and approved the submitted version.

## Funding

This study was supported by the Key Subject Construction Project of Shanghai Municipal Health Commission, No. shslczdzk04001.

## Conflict of interest

The authors declare that the research was conducted in the absence of any commercial or financial relationships that could be construed as a potential conflict of interest.

## Publisher's note

All claims expressed in this article are solely those of the authors and do not necessarily represent those of their affiliated organizations, or those of the publisher, the editors and the reviewers. Any product that may be evaluated in this article, or claim that may be made by its manufacturer, is not guaranteed or endorsed by the publisher.
