# Supplementary material for: Effectiveness of therapeutic massage for improving motor symptoms in Parkinson's disease: A systematic review and meta-analysis
Source: Front Neurol. 2022 Sep 5;13:915232. doi: 10.3389/fneur.2022.915232 (PMC9483027; doi:10.3389/fneur.2022.915232)
Supplement: Supplementary file 1 [file Data_Sheet_1.docx]

**TABLE 1** Searching terms for PubMed

| **Search** | **Query** | **Results** | **Date** |
| --- | --- | --- | --- |
| #1 | Massage [All Fields] | 17,515 | Jun 25, 2022 |
| #2 | Massage therapy [All Fields] | 16,944 | Jun 25, 2022 |
| #3 | Therapeutic massage [All Fields] | 16,944 | Jun 25, 2022 |
| #4 | Zone therapy [All Fields] | 36,792 | Jun 25, 2022 |
| #5 | Manual therapy [All Fields] | 45,453 | Jun 25, 2022 |
| #6 | Manipulative therapy [All Fields] | 19,949 | Jun 25, 2022 |
| #7 | Manipulation therapy [All Fields] | 35,027 | Jun 25, 2022 |
| #8 | Therapeutic manipulation [All Fields] | 16,944 | Jun 25, 2022 |
| #9 | Reflexology [All Fields] | 18,594 | Jun 25, 2022 |
| #10 | Musculoskeletal manipulation [All Fields] | 18,319 | Jun 25, 2022 |
| #11 | Tuina [All Fields] | 2,118 | Jun 25, 2022 |
| #12 | Acupressure [All Fields] | 1,640 | Jun 25, 2022 |
| #13 | #1 OR #2 OR #3 OR #4 OR #5 OR #6 OR #7 OR #8 OR #9 OR #10 OR #11 OR #12 | 127,110 | Jun 25, 2022 |
| #14 | Parkinson’s disease [All Fields] | 131,687 | Jun 25, 2022 |
| #15 | Parkinson disease [All Fields] | 131,687 | Jun 25, 2022 |
| #16 | Parkinsonism [All Fields] | 155,919 | Jun 25, 2022 |
| #17 | Primary Parkinsonism [All Fields] | 132,298 | Jun 25, 2022 |
| #18 | Idiopathic Parkinson’s disease [All Fields] | 131,655 | Jun 25, 2022 |
| #19 | Idiopathic Parkinson disease [All Fields] | 131,655 | Jun 25, 2022 |
| #20 | Lewy body Parkinson’s disease [All Fields] | 131,655 | Jun 25, 2022 |
| #21 | Paralysis agitans [All Fields] | 131,694 | Jun 25, 2022 |
| #22 | #14 OR #15 OR #16 OR #17 OR #18 OR #19 OR #20 OR #21 | 155,953 | Jun 25, 2022 |
| #23 | #13 AND #22 | 1,188 | Jun 25, 2022 |
| #24 | #23 Filters: Clinical Trial | 92 | Jun 25, 2022 |

| **Search** | **Query** | **Results** | **Date** |
| --- | --- | --- | --- |
| #1 | ‘Massage’ | 26,798 | Jun 25, 2022 |
| #2 | ‘Massage therapy’ | 1,799 | Jun 25, 2022 |
| #3 | ‘Therapeutic massage’ | 258 | Jun 25, 2022 |
| #4 | ‘Zone therapy’ | 58 | Jun 25, 2022 |
| #5 | ‘Manual therapy’ | 5,828 | Jun 25, 2022 |
| #6 | ‘Manipulative therapy’ | 1,965 | Jun 25, 2022 |
| #7 | ‘Manipulation therapy’ | 251 | Jun 25, 2022 |
| #8 | ‘Therapeutic manipulation’ | 1,352 | Jun 25, 2022 |
| #9 | ‘Reflexology’ | 1,307 | Jun 25, 2022 |
| #10 | ‘Musculoskeletal manipulation’ | 242 | Jun 25, 2022 |
| #11 | ‘Tuina’ | 2,619 | Jun 25, 2022 |
| #12 | ‘Acupressure’ | 2,829 | Jun 25, 2022 |
| #13 | #1 OR #2 OR #3 OR #4 OR #5 OR #6 OR #7 OR #8 OR #9 OR #10 OR #11 OR #12 | 40,529 | Jun 25, 2022 |
| #14 | ‘Parkinson disease’ | 183,961 | Jun 25, 2022 |
| #15 | ‘Parkinsonism’ | 51,620 | Jun 25, 2022 |
| #16 | ‘Primary Parkinsonism’ | 37 | Jun 25, 2022 |
| #17 | ‘Idiopathic Parkinson disease’ | 563 | Jun 25, 2022 |
| #18 | ‘Lewy body Parkinson disease’ | 4 | Jun 25, 2022 |
| #19 | ‘Paralysis agitans’ | 1,024 | Jun 25, 2022 |
| #20 | #14 OR #15 OR #16 OR #17 OR #18 OR #19 | 211,527 | Jun 25, 2022 |
| #21 | #13 AND #20 | 225 | Jun 25, 2022 |
| #22 | #21 AND 'clinical trial'/de | 38 | Jun 25, 2022 |

**TABLE 2** Searching terms for Embase

| **Search** | **Query** | **Results** | **Date** |
| --- | --- | --- | --- |
| #1 | (Massage) | 6,500 | Jun 25, 2022 |
| #2 | (Massage therapy) | 884 | Jun 25, 2022 |
| #3 | (Therapeutic massage) | 1,117 | Jun 25, 2022 |
| #4 | (Zone therapy) | 2,087 | Jun 25, 2022 |
| #5 | (Manual therapy) | 13,587 | Jun 25, 2022 |
| #6 | (Manipulative therapy) | 1,907 | Jun 25, 2022 |
| #7 | (Manipulation therapy) | 3,944 | Jun 25, 2022 |
| #8 | (Therapeutic manipulation) | 1,332 | Jun 25, 2022 |
| #9 | (Reflexology) | 650 | Jun 25, 2022 |
| #10 | (Musculoskeletal manipulation) | 627 | Jun 25, 2022 |
| #11 | (Tuina) | 401 | Jun 25, 2022 |
| #12 | (Acupressure) | 1,812 | Jun 25, 2022 |
| #13 | #1 OR #2 OR #3 OR #4 OR #5 OR #6 OR #7 OR #8 OR #9 OR #10 OR #11 OR #12 | 26,937 | Jun 25, 2022 |
| #14 | (Parkinson’s disease) | 11,552 | Jun 25, 2022 |
| #15 | (Parkinson disease) | 11,555 | Jun 25, 2022 |
| #16 | (Parkinsonism) | 1,825 | Jun 25, 2022 |
| #17 | (Primary Parkinsonism) | 650 | Jun 25, 2022 |
| #18 | (Idiopathic Parkinson’s disease) | 1,252 | Jun 25, 2022 |
| #19 | (Idiopathic Parkinson disease) | 1,252 | Jun 25, 2022 |
| #20 | (Lewy body Parkinson’s disease) | 293 | Jun 25, 2022 |
| #21 | (Paralysis agitans) | 13 | Jun 25, 2022 |
| #22 | #14 OR #15 OR #16 OR #17 OR #18 OR #19 OR #20 OR #21 | 12,275 | Jun 25, 2022 |
| #23 | #13 AND #22 | 419 | Jun 25, 2022 |
| #24 | #23 in Trials | 160 | Jun 25, 2022 |

**TABLE 3** Searching terms for Cochrane Library

| **Search** | **Query** | **Results** | **Date** |
| --- | --- | --- | --- |
| #1 | 全文：（推拿） | 239,112 | Jun 25, 2022 |
| #2 | 全文：（按摩） | 734,458 | Jun 25, 2022 |
| #3 | 全文：（手法治疗） | 69,415 | Jun 25, 2022 |
| #4 | 全文：（正骨） | 93,579 | Jun 25, 2022 |
| #5 | 全文：（点穴） | 37,940 | Jun 25, 2022 |
| #6 | #1 OR #2 OR #3 OR #4 OR #5 | 955,765 | Jun 25, 2022 |
| #7 | 全文：（帕金森病） | 114,667 | Jun 25, 2022 |
| #8 | 全文：（帕金森氏病） | 20,711 | Jun 25, 2022 |
| #9 | 全文：（路易小体帕金森病） | 52 | Jun 25, 2022 |
| #10 | 全文：（特发性帕金森病） | 632 | Jun 25, 2022 |
| #11 | 全文：（帕金森综合征） | 17,906 | Jun 25, 2022 |
| #12 | 全文：（震颤性麻痹） | 1,309 | Jun 25, 2022 |
| #13 | #7 OR #8 OR #9 OR #10 OR #11 OR #12 | 138,909 | Jun 25, 2022 |
| #14 | #6 AND #13 | 7,902 | Jun 25, 2022 |
| #15 | #14 with （筛选项：临床研究） | 539 | Jun 25, 2022 |

**TABLE 4** Searching terms for CNKI

| Study | Country | Type | Gender | | Age (Mean ± SD) | | Duration (Mean ± SD) | | Severity |
| --- | --- | --- | --- | --- | --- | --- | --- | --- | --- |
|  |  |  | Male | Female | Experimental | Control | Experimental | Control |  |
| Xu2012 | China | RCT | 27 | 13 | 55.70±7.08 | 56.55±6.99 | 7.95±3.80 | 2.08±0.59 | H&Y II-III |
| Zeng2014 | China | RCT | 46 | 17 | 63.4±5.88 | 62.5±6.52 | 2.1±1.40 | 1.5±0.87 | H&Y I-III |
| Zhao2016 | China | RCT | 25 | 11 | 70.18±9.21 | 70.21±10.70 | N/A | N/A | H&Y II-IV |
| Miyahara2018 | Thailand | RCT | 33 | 27 | 66.37±7.32 | 64.10±10.83 | 8.53±4.73 | 9.17±7.76 | H&Y I-III |
| Li2019 | China | RCT | 41 | 33 | 65.42±8.17 | 64.37±7.24 | 3.28±1.47 | 3.15±1.13 | N/A |
| Chen2019 | China | RCT | 22 | 32 | 82.18±5.70 | 83.63±5.27 | 6.15±1.42 | 6.26±2.77 | H&Y II-III |
| Yuen2021 | China | Randomized pilot trial | 13 | 23 | 63.77±1.41 | 64.64±2.57 | 8.41±1.27 | 8.64±1.44 | H&Y I-III |

**TABLE 5** Characteristics of PD participants

| Study | Participants  (experimental/control) | Intervention  (control) | Intervention  (experimental) | Time length of per treatment  (mins) | Frequency of massage (per wk) | Duration of massge (wks) | Follow-up (wks) | Primary outcome |
| --- | --- | --- | --- | --- | --- | --- | --- | --- |
| Xu2012 | 40 (20/20) | Health education | Traditional Chinese Tuina | N/A | N/A | 8 | instant | UPDRS-III |
| Zeng2014 | 63 (32/31) | Madopar | Traditional Chinese Tuina | 30 | 7 | 12 | instant | UPDRS-III |
| Zhao2016 | 36 (22/14) | Basic drugs (no detail) | Massage with thumbs and thenar eminence | 30 | 5 | 52 | instant | UPDRS-III |
| Miyahara2018 | 60 (30/30) | standard medical care | Therapeutic Thai massage | 20 | 2 | 3 | instant | UPDRS-III |
| Li2019 | 74 (37/37) | Madopar and pramipexole | Traditional Chinese Tuina | 36 | 7 | 4 | instant | UPDRS-III |
| Chen2019 | 54 (27/27) | Basic clinical care | Massage on limbs and face | 20 | 3 | 8 | 8 | UPDRS-III |
| Yuen2021 | 36 (14/22) | Health education | Self-acupressure | 60 | 7 | 8 | instant | UPDRS-III |

**TABLE 6** Characteristics of included studies intervention
